# Supplementary material for: Genomic Regions and Candidate Genes Linked to Capped Hock in Pig
Source: Life (Basel). 2021 May 31;11(6):510. doi: 10.3390/life11060510 (PMC8228005; doi:10.3390/life11060510)
Supplement: Supplementary file 1 [file life-11-00510-s001.zip › life-1213343-supplementary.pdf]

life

MDPI

Article

Genomic Regions and Candidate Genes Linked to Capped Hock in Pig

Lyubov Getmantseva [1,*], Maria Kolosova [1,2], Faridun Bakoev [1], Anna Zimina [1,*], and Siroj Bakoev [1,3]

[1] Federal Research Center for Animal Husbandry named after Academy Member L.K. Ernst, Dubrovitsy 142132, Russia; m.leonovaa@mail.ru (M.K.) bakoevfaridun@yandex.ru (F.B.); siroj1@yandex.ru (S.B.)
[2] Department of Biotechnology, Don State Agrarian University, Persianovski 346493, Russia
[3] Centre for Strategic Planning and Management of Biomedical Health Risks, Moscow 123182, Russia
* Correspondence: ilonaluba@mail.ru (L.G.); filipchenko-90@mail.ru (A.Z.); Tel.: +7-(4967)-65-11-01 (L.G. and A.Z.)

Abstract: Capped hock affects the exterior of pedigree pigs, making them unsalable and resulting in a negative impact on the efficiency of pig-breeding centers. The purpose of this paper was to carry out pilot studies aimed at finding genomic regions and genes linked to the capped hock in pigs. The studies were carried out on Landrace pigs (n = 75) and Duroc pigs (n = 70). To identify genomic regions linked to capped hock in pigs, we used smoothing FST statistics. Genotyping was performed with GeneSeek® GGP Porcine HD Genomic Profiler v1 (Illumina Inc, USA). The research results showed 70 SNPs linked to capped hock in Landrace (38 SNPs) and Duroc (32 SNPs). The identified regions overlapped with QTLs related with health traits (blood parameters) and meat and carcass traits (fatness). In total, 31 genes were identified (i.e., 17 genes in Landrace, 14 genes in Durocs). Three genes appeared in both the Landrace and Duroc groups, including A2ML1 (SSC5), ROBO2 (SSC13), and MSI1 (SSC14). We identified genomic regions directly or indirectly linked to capped hock, which thus might contribute to identifying genetic variants and using them as genetic markers in pig breeding.

Keywords: pig; capped hock; selection signatures; genome; candidate genes; A2ML1; ROBO2; MSI1

Citation: Getmantseva, L.; Kolosova, M.; Bakoev, F.; Zimina, A.; Bakoev, S. Genomic Regions and Candidate Genes Linked to Capped Hock in Pig. *Life* **2021**, *11*, 510. https://doi.org/10.3390/life11060510

Academic Editors: Yuriy Lvovich Orlov and Anastasia A. Anashkina

Received: 22 April 2021
Accepted: 28 May 2021
Published: 31 May 2021

Publisher's Note: MDPI stays neutral with regard to jurisdictional claims in published maps and institutional affiliations.

Copyright: © 2021 by the authors. Licensee MDPI, Basel, Switzerland. This article is an open access article distributed under the terms and conditions of the Creative Commons Attribution (CC BY) license (http://creativecommons.org/licenses/by/4.0/).

Supplementary Materials:

Table S1. SNPs with the most significant signals in L_I vs L_II.

Uploaded Variation	Chr	Location	SYMBOL	Existing Variation
MARC0030993	1	17549870	SASH1	rs80969276
ALGA0103472	2	39626373	NAV2	rs81330475
WU_10.2_2_128186090	2	122874593	DTWD2	rs334355498
WU_10.2_2_146797370	2	141009508	SIL1	rs320190225
MARC0073571	4	119421277	-	rs80849125
WU_10.2_5_65456803	5	62626025	A2ML1	rs341331983
ALGA0041880	7	49887262	-	rs80994093
ASGA0038682	8	36628170	-	rs81399897
DRGA0008554	8	36640393	-	rs81301907
ASGA0038686	8	36694976	-	rs81399904
MARC0083335	9	85218746	-	rs81266663
ASGA0051491	11	65796898	HS6ST3	rs80999276
MARC0003871	11	66242426	-	rs81227709
ASGA0091209	11	66385031	MBNL2	rs81477757
MARC0046077	12	33946887	MSI2	rs81237783
WU_10.2_12_41135707	12	39566229	-	rs318621721
ALGA0120101	13	178817874	ROBO2	rs81327419
ALGA0074279	14	1594168	-	rs80831536
WU_10.2_14_1958251	14	1707134	-	rs325491375
WU_10.2_14_5494122	14	5211273	-	rs335163579
WU_10.2_14_25998805	14	24451474	RIMBP2	rs338806883
ASGA0062222	14	24472907	RIMBP2	rs80931297
ALGA0076318	14	24580553	RIMBP2	rs80976116
MARC0079723	14	40329970	MSI1	rs81264077
ASGA0062993	14	41641323	-	rs80783712
ALGA0077159	14	41697208	-	rs80983909
ALGA0077175	14	41894877	SVOP	rs80863866
H3GA0039979	14	42273660	SART3	rs80827280
MARC0004842	14	42353262	-	rs80970794
ASGA0063071	14	42910896	SGSM1	rs80905649
ALGA0078910	14	74444995	CDH23	rs80965661
WU_10.2_15_143794446	15	129863001	-	rs334067331
SIRI0001312	15	130037634	-	rs318303466
MARC0090673	15	130082478	PID1	rs81272043
ASGA0071304	15	130450029	DNER	rs81455786
WU_10.2_17_8800657	17	7921242	-	rs321482623
WU_10.2_17_14603707	17	13757956	-	rs320374299
WU_10.2_17_47910100	17	42408936	-	rs341759761

SSC – Sus scrofa chromosome.

Table S2. QTLs, determined in L_I vs L_II.

ID QTL	SSC	Trait	Class	Group
15128	15	Bilirubin level	Blood parameters	Health Traits
16335	2	Backfat at tenth rib	Fatness	Meat and carcass trait
18087	14	Total number born alive	Litter traits	Reproduction traits
18128	5	Number of stillborn	Litter traits	Reproduction traits
22138	8	Mean corpuscular hemoglobin concentration	Blood parameters	Health Traits
22144	8	Mean corpuscular volume	Blood parameters	Health Traits
22145	8	Red blood cell count	Blood parameters	Health Traits
22147	8	Red blood cell count	Blood parameters	Health Traits
22162	8	Mean corpuscular hemoglobin concentration	Blood parameters	Health Traits
22164	8	Mean corpuscular volume	Blood parameters	Health Traits
22165	8	Red blood cell count	Blood parameters	Health Traits
22168	8	Platelet distribution width	Blood parameters	Health Traits
23157	17	Backfat thickness between 3rd and 4th rib	Fatness	Meat and carcass trait
23164	17	Backfat thickness between 3rd and 4th rib	Fatness	Meat and carcass trait
30994	15	Maternal infanticide	Behavioral	Exterior traits
31840	2	Corpus luteum number	Litter traits	Reproduction traits
32128	14	Linoleic acid content	Fatty acid content	Meat and carcass trait
64761	11	Spinal curvature	Defects	Exterior traits
65002	2	Heart weight	Anatomy	Meat and carcass trait
66275	1	Body weight	Growth	Production traits
95692	1	Ham weight	Anatomy	Meat and carcass trait
106221	2	Piglet mortality	Litter traits	Reproduction traits
121898	12	Meat color b*	Meat color	Meat and carcass trait
125483	9	Intramuscular fat content	Fatness	Meat and carcass trait
126142	2	Conformation score	Conformation	Exterior traits
126624	14	Teat number	Reproductive organ	Reproduction traits
126660	8	Teat number	Reproductive organ	Reproduction traits
126705	14	Teat number difference between sides	Reproductive organ	Reproduction traits
126735	14	Left teat number	Reproductive organ	Reproduction traits
140352	9	PRRSV susceptibility	Disease susceptibility	Health Traits
161332	15	Conductivity 45 minutes post-mortem	Conductivity impedance	Meat and carcass trait
161367	15	Hair amount	Coat gharacteristics	Exterior traits
161368	15	Hair amount	Coat gharacteristics	Exterior traits
161369	15	Hair amount	Coat gharacteristics	Exterior traits
161370	15	Hair amount	Coat gharacteristics	Exterior traits
167228	15	pH 24 hr post-mortem loin	pH	Meat and carcass trait
167229	15	Cooking yield	Texture	Meat and carcass trait
167230	15	Drip loss	Texture	Meat and carcass trait
167231	15	muscle protein percentage	Chimestry	Meat and carcass trait
170629	15	Average backfat thickness	Fatness	Meat and carcass trait
170646	15	Lean meat percentage	Fatness	Meat and carcass trait
170898	15	Days to 100 kg	Growth	Production traits
170991	14	Basophil number	Immune capacity	Health Traits
170992	14	Basophil number	Immune capacity	Health Traits
170993	14	Basophil number	Immune capacity	Health Traits
170994	14	Basophil number	Immune capacity	Health Traits
170995	14	Basophil number	Immune capacity	Health Traits
170996	14	Basophil number	Immune capacity	Health Traits
170997	14	Basophil number	Immune capacity	Health Traits
170998	14	Basophil number	Immune capacity	Health Traits
170999	14	Basophil number	Immune capacity	Health Traits
171000	14	Basophil number	Immune capacity	Health Traits
171001	14	Basophil number	Immune capacity	Health Traits

ID QTL	SSC	Trait	Class	Group
171002	14	Basophil number	Immune capacity	Health Traits
171003	14	Basophil number	Immune capacity	Health Traits
171004	14	Basophil number	Immune capacity	Health Traits
171005	14	Basophil number	Immune capacity	Health Traits
171006	14	Basophil number	Immune capacity	Health Traits
171007	14	Basophil number	Immune capacity	Health Traits
171008	14	Basophil number	Immune capacity	Health Traits
171009	14	Basophil number	Immune capacity	Health Traits
171010	14	Basophil number	Immune capacity	Health Traits
171011	14	Basophil number	Immune capacity	Health Traits
171012	14	Basophil number	Immune capacity	Health Traits
171013	14	Basophil number	Immune capacity	Health Traits
171014	14	Basophil number	Immune capacity	Health Traits
171015	14	Basophil number	Immune capacity	Health Traits
171016	14	Basophil number	Immune capacity	Health Traits
171017	14	Basophil number	Immune capacity	Health Traits
171018	14	Basophil number	Immune capacity	Health Traits
171019	14	Basophil number	Immune capacity	Health Traits
171020	14	Basophil number	Immune capacity	Health Traits
171021	14	Basophil number	Immune capacity	Health Traits
171022	14	Basophil number	Immune capacity	Health Traits
171023	14	Basophil number	Immune capacity	Health Traits
171024	14	Basophil number	Immune capacity	Health Traits
171025	14	Basophil number	Immune capacity	Health Traits
171026	14	Basophil number	Immune capacity	Health Traits
171027	14	Basophil number	Immune capacity	Health Traits
171028	14	Basophil number	Immune capacity	Health Traits
171029	14	Basophil number	Immune capacity	Health Traits
171030	14	Basophil number	Immune capacity	Health Traits
171031	14	Basophil number	Immune capacity	Health Traits
171032	14	Basophil number	Immune capacity	Health Traits
171033	14	Basophil number	Immune capacity	Health Traits
171034	14	Basophil number	Immune capacity	Health Traits
171035	14	Basophil number	Immune capacity	Health Traits
171036	14	Basophil number	Immune capacity	Health Traits
171037	14	Basophil number	Immune capacity	Health Traits
171038	14	Basophil number	Immune capacity	Health Traits
171039	14	Basophil number	Immune capacity	Health Traits
171040	14	Basophil number	Immune capacity	Health Traits
171041	14	Basophil number	Immune capacity	Health Traits
171042	14	Basophil number	Immune capacity	Health Traits
171043	14	Basophil number	Immune capacity	Health Traits
171044	14	Basophil number	Immune capacity	Health Traits
171045	14	Basophil number	Immune capacity	Health Traits
171046	14	Basophil number	Immune capacity	Health Traits
171047	14	Basophil number	Immune capacity	Health Traits
171048	14	Basophil number	Immune capacity	Health Traits
171049	14	Basophil number	Immune capacity	Health Traits
171050	14	Basophil number	Immune capacity	Health Traits
171051	14	Basophil number	Immune capacity	Health Traits
171052	14	Basophil number	Immune capacity	Health Traits
171053	14	Basophil number	Immune capacity	Health Traits
171054	14	Basophil number	Immune capacity	Health Traits
171055	14	Basophil number	Immune capacity	Health Traits
171056	14	Basophil number	Immune capacity	Health Traits
171057	14	Basophil number	Immune capacity	Health Traits

ID QTL	SSC	Trait	Class	Group
171058	14	Basophil number	Immune capacity	Health Traits
171059	14	Basophil number	Immune capacity	Health Traits
171060	14	Basophil number	Immune capacity	Health Traits
171061	14	Basophil number	Immune capacity	Health Traits
171062	14	Basophil number	Immune capacity	Health Traits
171063	14	Basophil number	Immune capacity	Health Traits
171064	14	Basophil number	Immune capacity	Health Traits
171065	14	Basophil number	Immune capacity	Health Traits
171066	14	Basophil number	Immune capacity	Health Traits
171067	14	Basophil number	Immune capacity	Health Traits
171068	14	Basophil number	Immune capacity	Health Traits
171069	14	Basophil number	Immune capacity	Health Traits
171070	14	Basophil number	Immune capacity	Health Traits
171071	14	Basophil number	Immune capacity	Health Traits
171072	14	Basophil number	Immune capacity	Health Traits
171073	14	Basophil number	Immune capacity	Health Traits
171074	14	Basophil number	Immune capacity	Health Traits
171075	14	Basophil number	Immune capacity	Health Traits
171090	14	LDL cholesterol	Blood parameters	Health Traits
171093	14	Mean corpuscular hemoglobin content	Blood parameters	Health Traits
171095	14	Mean corpuscular hemoglobin concentration	Blood parameters	Health Traits
171103	14	Blood non-esterified fatty acid level	Blood parameters	Health Traits
171104	14	Blood non-esterified fatty acid level	Blood parameters	Health Traits
171105	14	Blood non-esterified fatty acid level	Blood parameters	Health Traits
171106	14	Blood non-esterified fatty acid level	Blood parameters	Health Traits
171107	14	Blood non-esterified fatty acid level	Blood parameters	Health Traits
171108	14	Blood non-esterified fatty acid level	Blood parameters	Health Traits
171109	14	Blood non-esterified fatty acid level	Blood parameters	Health Traits
171110	14	Blood non-esterified fatty acid level	Blood parameters	Health Traits
171111	14	Blood non-esterified fatty acid level	Blood parameters	Health Traits
171120	14	Bilirubin level	Blood parameters	Health Traits
171144	5	Blood urea level	Blood parameters	Health Traits
178883	14	Number of mummified pigs	Litter traits	Reproduction traits
178884	14	Number of mummified pigs	Litter traits	Reproduction traits
178885	14	Number of mummified pigs	Litter traits	Reproduction traits
179299	14	Litter weight total	Litter traits	Reproduction traits
179305	14	Litter size	Litter traits	Reproduction traits
179306	14	Litter size	Litter traits	Reproduction traits

SSC – Sus scrofa chromosome.

Table S3. SNPs with the most significant signals in D_I vs D_II.

Uploaded_Variation	SSC	Location	SYMBOL	Existing_Variation
WU_10.2_10_6743776	10	5059529	-	rs81427537
WU_10.2_11_5321086	11	5634464	FLT1	rs324295783
MARC0046077	12	33946887	MSI2	rs81237783
WU_10.2_13_9673699	13	8427269	ZNF385D	rs327907552
WU_10.2_13_9726957	13	8480553	ZNF385D	rs332080276
WU_10.2_13_9767339	13	8520901	ZNF385D	rs328536473
WU_10.2_13_9782147	13	8535711	ZNF385D	rs319396990
WU_10.2_13_9873963	13	8628750	-	rs341648836
WU_10.2_13_9919470	13	8690551	-	rs331208680
ALGA0116253	13	23544359	SCN10A	rs81478668
ALGA0113417	13	23581312	-	rs81342178
WU_10.2_13_25818975	13	23600960	SCN11A	rs346314104
ASGA0100028	13	23669797	SCN11A	rs81478395
WU_10.2_13_25953293	13	23693721	SCN11A	rs339492216
H3GA0035852	13	23741303	WDR48	rs81443702
ASGA0056686	13	23765878	WDR48	rs81286678
ASGA0056707	13	23800213	TTC21A	rs81278459
ALGA0068887	13	23857751	-	rs81443711
ALGA0120101	13	178817874	ROBO2	rs81327419
WU_10.2_14_5494122	14	5211273	-	rs335163579
WU_10.2_16_6524638	16	6079228	MYO10	rs338457670
ALGA0090202	16	33611061	-	rs81458369
ALGA0018559	3	36808586	RBFOX1	rs81369266
WU_10.2_3_126402455	3	118364336	-	rs80881141
WU_10.2_5_65456803	5	62626025	A2ML1	rs341331983
ALGA0041880	7	49887262	-	rs80994093
WU_10.2_7_102488461	7	96694364	NUMB	rs329434246
WU_10.2_8_29947191	8	28581006	-	rs339146187
MARC0083335	9	85218746	-	rs81266663
WU_10.2_9_120099467	9	109160356	-	rs341080205
WU_10.2_9_140916040	9	128362353	KCNK2	rs326736106
WU_10.2_9_144258473	9	131211605	DTL	rs326767944

SSC – Sus scrofa chromosome.

Table S4. QTLs, determined in D_I vs D_II.

ID QTL	SSC	Trait	Class	Group
18128	5	Number of stillborn	Litter traits	Reproduction traits
18134	13	Number of stillborn	Litter traits	Reproduction traits
18648	3	androstenone laboratory	Chemistry	Meat and carcass trait
29611	9	Body weight weaning	Growth	Production traits
32075	8	Palmitic acid content	Fatty acid content	Meat and carcass trait
32128	14	Linoleic acid content	Fatty acid content	Meat and carcass trait
55880	11	Cryptorchidism	Reproductive organ	Reproduction traits
55881	11	Cryptorchidism	Reproductive organ	Reproduction traits
95388	16	Arachidic acid content	Fatty acid content	Meat and carcass trait
107244	16	Hematocrit	Blood parameters	Health Traits
107323	16	Red blood cell count	Blood parameters	Health Traits
121898	12	Meat color b*	Meat color	Meat and carcass trait
125483	9	Intramuscular fat content	Fatness	Meat and carcass trait
126115	3	Top line conformation	Conformation	Exterior traits
126657	7	Left teat number	Reproductive organ	Reproduction traits
126682	7	Teat number	Reproductive organ	Reproduction traits
135646	9	Daily feed intake	Feed intake	Production traits
139179	10	Lean meat percentage	Fatness	Meat and carcass trait
139189	9	Backfat between 3rd and 4th last ribs	Fatness	Meat and carcass trait
140352	9	PRRSV susceptibility	Disease susceptibility	Health Traits
147209	3	Cannon bone circumference	Conformation	Exterior traits
147212	3	Chest circumference	Growth	Production traits
147273	3	Intramuscular fat content	Fatness	Meat and carcass trait
147274	3	Intramuscular fat content	Fatness	Meat and carcass trait
147275	3	Intramuscular fat content	Fatness	Meat and carcass trait
147276	3	Intramuscular fat content	Fatness	Meat and carcass trait
147277	3	Intramuscular fat content	Fatness	Meat and carcass trait
147278	3	Intramuscular fat content	Fatness	Meat and carcass trait
147279	3	Intramuscular fat content	Fatness	Meat and carcass trait
147280	3	Intramuscular fat content	Fatness	Meat and carcass trait
147281	3	Intramuscular fat content	Fatness	Meat and carcass trait
147282	3	Intramuscular fat content	Fatness	Meat and carcass trait
147283	3	Intramuscular fat content	Fatness	Meat and carcass trait
147284	3	Intramuscular fat content	Fatness	Meat and carcass trait
147285	3	Intramuscular fat content	Fatness	Meat and carcass trait
147286	3	Intramuscular fat content	Fatness	Meat and carcass trait
147287	3	Intramuscular fat content	Fatness	Meat and carcass trait
147288	3	Intramuscular fat content	Fatness	Meat and carcass trait
147289	3	Intramuscular fat content	Fatness	Meat and carcass trait
147290	3	Intramuscular fat content	Fatness	Meat and carcass trait
147291	3	Intramuscular fat content	Fatness	Meat and carcass trait
147292	3	Intramuscular fat content	Fatness	Meat and carcass trait
147293	3	Intramuscular fat content	Fatness	Meat and carcass trait
164851	16	pH 24 hr post-mortem loin	pH	Meat and carcass trait
167224	11	Dressing percentage	Anatomy	Meat and carcass trait
167225	7	Number of ribs	Anatomy	Meat and carcass trait
171144	5	Blood urea level	Blood parameters	Health Traits
171149	7	Hemoglobin	Blood parameters	Health Traits
179299	14	Litter weight total	Litter traits	Reproduction traits
